# Side Effects of Treating Acne Vulgaris With Isotretinoin: A Systematic Review

**DOI:** 10.7759/cureus.55946

**Published:** 2024-03-11

**Authors:** Ishika Rajput, Vaibhav P Anjankar

**Affiliations:** 1 Medical School, Jawaharlal Nehru Medical College, Datta Meghe Institute of Higher Education & Research, Wardha, IND; 2 Anatomy, Jawaharlal Nehru Medical College, Datta Meghe Institute of Higher Education & Research, Wardha, IND

**Keywords:** gastrointestinal, dosage, psychological, treatment, adverse reactions, side effects, isotretinoin, acne vulgaris

## Abstract

The treatment of acne vulgaris poses a significant challenge due to its chronic nature and potential influence on patients’ quality of life. Isotretinoin, a systemic retinoid, has emerged as one of the most efficient treatment options for chronic, severe acne. However, the use of isotretinoin is associated with a range of side effects that require careful consideration. This review article provides a comprehensive overview of the side effects linked to isotretinoin treatment for acne vulgaris. Through an analysis of existing literature and clinical studies, we discuss the various adverse reactions, their incidence, management strategies, and the influence of these side effects on patients’ quality of life.

## Introduction and background

Acne is ranked the eighth most prevalent illness worldwide, affecting around 9.4% of the population [1]. Scarring occurs due to acne, and especially severe acne-related scarring may last a lifetime and cause long-lasting psychosocial repercussions [2]. So, among the desired outcomes of treatment are lesion resolution, psychological morbidity reduction, and scar prevention [3]. A rise in the production of sebum, altered keratinization, *Propionibacterium acnes* (now known as *Cutibacterium acnes*) bacteria colonizing the hair follicles primarily on the face but also on the neck, chest, and back, and an inflammatory reaction of the skin are all associated with androgen [2]. Topical creams or ointments can treat mild cases of acne vulgaris. However, systemic therapy will be necessary to treat the disease’s severe manifestations [4].

Isotretinoin, a retinoid authorized by the FDA in 1982, is one of the most efficient treatments for acne [5]. Reduced keratinization, reduction of the inflammatory response, and limited inhibition of sebaceous gland activity are the impacts of isotretinoin on acne vulgaris [6]. The only medication that can treat all of the pathogenic causes of acne is isotretinoin [6]. Despite the fact that isotretinoin is quite successful in treating acne vulgaris, the increasing frequency of adverse effects severely restricts its use [4]. The most notable effect is its teratogenicity [7]. Other documented side effects are mucocutaneous, ophthalmic, and musculoskeletal [8]. In addition to these adverse effects, isotretinoin is known to produce a number of lab abnormalities, which include effects on the levels of plasma lipids and liver function tests [9]. Hematological profile disturbances are yet another significant side effect [4]. Therefore, it is advised to establish a baseline total blood count and to regularly check for any changes in blood count while treating acne vulgaris [4].

Isotretinoin is a drug that is used very widely around the world for the treatment of acne vulgaris, yet often medical professionals fail to inform patients about the possible side effects. This is why we feel the need to write this article. We will discuss these side effects in further detail in this article.

## Review

Methodology

**Figure 1:**
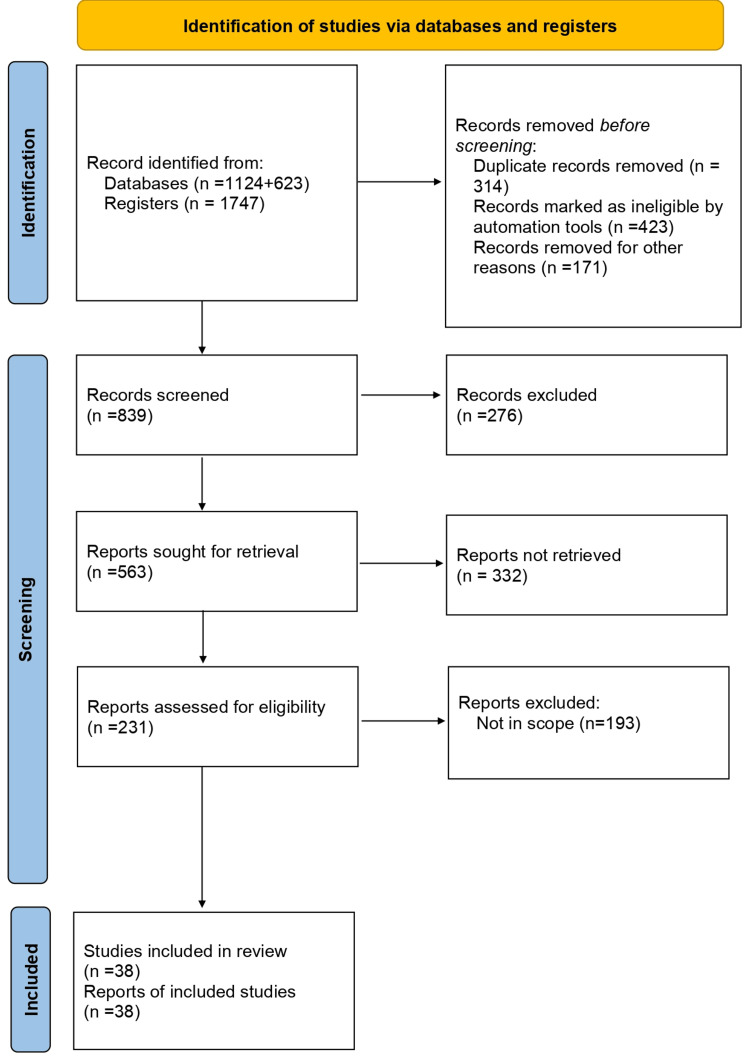
The selection process of the articles used in this study Adopted from the Preferred Reporting Items for Systematic Reviews and Meta-Analyses (PRISMA)

We reviewed PubMed, Cureus, and a few other open-access journal articles on isotretinoin and its varying side effects. We searched for and included as many relevant studies as possible using medical subject heading phrases like “treating acne vulgaris with isotretinoin” and “side effects of isotretinoin,” as well as various keyword combos like isotretinoin, acne vulgaris, psychological effects, and treatment. To identify potential additional records from other sources, a literature search was conducted to locate case-control studies and meta-analyses (Figure 1).

Side effects of isotretinoin

Mucocutaneous Side Effects 

Nearly all patients who get treatment have skin xerosis, particularly on skin that is exposed, and cheilitis as the first and most common side effects [10]. Due to the paucity of discussion in the literature, doctors need a more comprehensive understanding of the frequency, timing, dosage dependence, and recoverability of hair loss with isotretinoin therapy [11]. Another detrimental side effect that affects the patient both mentally and physically is facial breakouts that resemble seborrheic dermatitis, although this is quite an uncommon side effect [12].

Musculoskeletal Side Effects

Myalgia, stiffness in muscles, true myopathy, or rhabdomyolysis are the adverse musculoskeletal effects associated with oral isotretinoin [10]. One of the widespread negative effects of isotretinoin is lower back pain [13]. Following isotretinoin therapy, sacroiliitis has been noted as an uncommon side effect [14]. Lower back pain triggered by isotretinoin is dosage dependent, and inflammatory back pain without sacroiliitis is also frequent [13]. The exact mechanism of retinoid-induced sacroiliitis is still not clear, but it has been proposed that isotretinoin alters the lysosomal membrane structure of cells, rendering the synovial cells more vulnerable to degeneration and minor injuries [14]. With radiographic characteristics like diffuse idiopathic skeletal hyperostosis, the development of skeletal hyperostosis has been linked to isotretinoin [15]. It has been demonstrated that long-term isotretinoin usage causes hyperostosis to spread and increase in number, deteriorating radiographically [15]. While receiving treatment, mild, non-specific musculoskeletal symptoms have been observed; however, they are not associated with radiographic abnormalities [15]. Exercise and the use of systemic isotretinoin may both work together to cause rhabdomyolysis [16]. Despite being a known potential side effect of isotretinoin use, routine screening or monitoring is not currently advised for rhabdomyolysis, the reason being its rarity [16].

Gastrointestinal Side Effects

Controversially, isotretinoin has been associated with the emergence of inflammatory bowel disease (IBD) [17]. In individuals who are more prone to developing ulcerative colitis (UC), isotretinoin may do so [17]. Epithelial cell growth restriction and T-cell activation are suspected causes of isotretinoin-induced UC [17]. By changing the lipid profile (including high-density lipoprotein, HDL), isotretinoin may potentially cause the inflammatory process, leading to the manifestation of IBD [17]. Despite not being listed as a cause of IBD, isotretinoin has the capability to induce temporary enteric inflammation and damage, especially in the small intestine, in the absence of other factors [18].

Acute pancreatitis is a rarely documented, severe adverse effect [19]. Pancreatitis brought on by isotretinoin may be idiosyncratic or brought on by high levels of serum triglycerides (TGs) [19]. Contrary to pancreatitis triggered by elevated TGs, idiosyncratic has no recognizable pattern and, therefore, cannot be observed and is a rare occurrence [20]. Isotretinoin is classified under category III of drug-induced pancreatitis (DIP) (some supportive evidence is present, but it is not very consistent) and has been identified as a rare cause of DIP [20].

Hematological Side Effects

Decreased mean platelet volume and platelet counts were observed in patients receiving isotretinoin for acne vulgaris [6]. Although the impact of isotretinoin on platelets is not fully understood, the impact of IL-6 on platelet formation may contribute to isotretinoin-induced thrombocytosis [6]. Bone marrow suppression may be the cause of isotretinoin-induced thrombocytopenia [6].

Biochemical Side Effects

Serum TG, alanine transaminase, and aspartate transaminase values rose as a result of treating acne with oral isotretinoin [9]. The risk of developing metabolic syndrome may rise as a result of this rise in TG levels when taking isotretinoin [9]. A rise in low-density lipoprotein and TG levels, as well as a decline in HDL levels, was observed among treated individuals [21]. It is possible that a decrease in the rate at which lipids are removed from plasma is causing the rise in TG levels in patients receiving oral isotretinoin treatment [9].

Rising insulin resistance brought on by isotretinoin may be linked to elevated levels of lipid oxidation via the Randle cycle [22]. Isotretinoin-induced insulin resistance might clinically exhibit latent autoimmune diabetes in adults [22]. Isotretinoin capsules, a product containing soybean oil, are not advised in patients with a history of soy or peanut allergies (soy and peanut are connected phylogenetically and antigenetically and share multiple homologous proteins) due to the threat of cross-reactivity [23].

Psychiatric Side Effects

Depression symptoms were notably reduced as a result of isotretinoin use in acne patients [24]. The event of isotretinoin producing an idiosyncratic reaction resulting in severe depression and suicidal tendencies, as proposed by some research papers, is still a prospect, albeit, if so, this seems to be a relatively rare incident [8]. There was no proof to suggest that isotretinoin increased the likelihood of newly diagnosed depression, other psychological conditions, or suicidal ideation in patients who were already not suffering from some other kind of mental disorder [25].

Severe insomnia has been linked to the utilization of isotretinoin for treating moderate to severe acne vulgaris; as a result, patients susceptible to insomnia are prone to develop mood disorders such as depression and psychosis [26]. Insomnia could be the initial symptom of a drug-induced psychiatric disorder [26]. In research conducted by Suuberg in 2019, a patient with insomnia who began using isotretinoin two months later experienced acute psychosis, which subsided after stopping the medication [27]. This emphasizes the significance of paying close attention to individuals who have insomnia as an initial symptom after starting isotretinoin [27].

Schizophrenia has previously been associated with retinoid dysregulation [27]. The risk of mood disorders, such as suicidal thoughts, increases in bipolar patients taking isotretinoin [27].

By lowering adult neurogenesis and altering the serotonergic neurotransmitter system’s constituents, isotretinoin inhibits serotonin signaling [28]. Patients on isotretinoin have been shown to have a decreased orbitofrontal cortex metabolism via functional brain imaging [28]. The presented effects of isotretinoin on the limbic system regions and cortical zones associated with bipolar disorder (BPD), the orbitofrontal cortex regions associated with affective and psychotic illnesses, and the orbitofrontal cortex regions linked with BPD may contribute to the emergence of isotretinoin-related psychotic mania [28].

Other Side Effects 

Patients consuming oral isotretinoin experienced headaches and taste/smell loss less frequently when infected with COVID-19 as compared to those who were not on the medication [29]. According to olfactory function tests conducted before and after starting oral isotretinoin therapy, it is observed that oral isotretinoin improves humans’ sense of smell [29].

The results of a study showed that isotretinoin may be helpful for some Cushing’s disease patients, specifically those with mild hypercortisolism [30]. According to the same study, approximately 44% of patients experienced the typical side effects of the medication, though they were generally mild and transient; these included conjunctival irritation, cheilitis, nausea, headaches, and arthralgias [30].

The primary chemopreventive and anticancer effects of the drug are due to isotretinoin-induced apoptosis [10]. In the tumor cells that release adrenocorticotropin, retinoic acid suppresses cell proliferation and triggers apoptosis [30].

Teratogenic Effects

Pregnancy is an absolute contraindication to isotretinoin as it is linked to various congenital disabilities, which include anomalies of the neurological system, the heart, and the craniofacial region [27]. Several distinct leptomeningeal neuroglial heterotopias, hydrocephalus, anomalies of the corticospinal tracts, mid-hindbrain anomalies, craniofacial deformities like anotia and microtia, anomalies related to the inner ear, anomalies of the eye, retina, or optic nerve, which include myopia and light sensitivity, psychomotor retardation, mental retardation, learning disabilities, agenesis of the cerebellar vermis, abnormal formation of the posterior fossa, and premature birth are the reported birth defects associated with isotretinoin [27].

Rare Side Effects

Premature growth plate fusion triggered by isotretinoin seemed related to a wide range of dosages and periods ranging from months to years [31]. Isotretinoin may disrupt the proximal tibia and distal femur’s growth plates [31].

Isotretinoin prevented the rise in blood pressure and markedly decreased albuminuria in rats with Thy1.1 glomerulonephritis [32]. Isotretinoin therapy also markedly decreased the number of glomerular and interstitial macrophages [32]. As demonstrated by many functional and histological markers, isotretinoin treatment substantially decreases glomerular and interstitial damage in rats suffering from chronic glomerulonephritis [32]. A unique therapeutic alternative for treating glomerulonephritis may be that of retinoids [32].

Cases of pseudotumor cerebri and herpes encephalitis, although rare, are reported due to isotretinoin use [33]. Increased intracranial pressure may be a side effect of isotretinoin [33]. Obese young women are more susceptible to pseudotumor cerebri [33]. Isotretinoin may be linked with numerous cardiac side effects, which include Kounis syndrome [34].

Impact on quality of life

The adverse effects of the acne condition on the patient’s overall quality of life were found to have significantly decreased after the treatment [35]. Physical function, vitality, social function, and mental health all showed substantial improvement (p < 0.001) [35]. Additionally, there was a substantial improvement in overall health and emotional role function (p < 0.005) [35].

Dosage of isotretinoin

For extreme cases of treatment-resistant acne, the typical dosage is 0.5-1.0 mg/kg every day for around 20 weeks, or a total of 120 mg/kg [3]. With a starting dose of 0.5 mg or less per kg daily, the severity of the first flare-ups can be reduced [3]. Less than 120 mg in total cumulative doses raises relapse rates, whereas more than 150 mg raises the probability of side effects without increasing benefits [3]. With a 120-mg cumulative dose, roughly 40% of patients acquire long-term clearance, 40% need topical therapies or oral antibiotics to be restarted, and 20% need isotretinoin to be restarted [3]. Low doses (0.3 mg/kg/per day) may be effective for those with moderate acne, and they will likely experience fewer side effects [3]. Isotretinoin dosages of 0.5 mg/kg/day cause hair loss at a frequency of 3.2%, and 0.5 mg/kg/day causes hair loss at a frequency of 5.7%, respectively [11]. Oral isotretinoin therapy is dose dependent; therefore, it is essential to investigate a person’s genetic affinity for tolerating a particular dosage [36].

Risk factors

A study shows that sebocyte apoptosis is isotretinoin’s primary mechanism of action, which may lead to genetic variation; the increased risk of side effects or treatment resistance in specific patient subgroups may be explained by these genetic variations of key regulators of isotretinoin-induced apoptotic signaling [10]. Another study examined the relationship between the side effects and age, gender, dose of isotretinoin (mg/kg/day), treatment period, and isotretinoin-naiveness; it showed that there was no connection between them [37]. The study investigated the following adverse effects: fatigue, myalgia, and low back pain [37].

Precautions and management

For patients suffering from acne vulgaris on isotretinoin, oral omega-3 cuts down on the mucocutaneous adverse effects of the medication [38]. In order to enable immediate control of mood attacks in risk groups, it is essential that acne treatment be carried out in coordination with dermatology and psychiatry professionals in patients with a personal or family history of psychological disease [28]. Cases without known risk factors should also be kept on the lookout for signs of mania and other psychiatric problems, and when and if needed, they should be immediately referred to the psychiatry department for a diagnosis and prompt treatment [28].

## Conclusions

Despite the possibility of adverse effects, isotretinoin therapy is still unrivaled in its ability to cure severe acne vulgaris. The decision to begin isotretinoin therapy is frequently motivated by the desire to see a significant improvement in the symptoms associated with acne vulgaris, which can have substantial psychosocial impacts of its own. Healthcare professionals must thoroughly evaluate patients for risk factors, provide them with information regarding possible adverse reactions, and advise the patient to stop the medication if they notice any of the side effects. Healthcare professionals may maximize treatment outcomes while minimizing suffering and improving patients’ quality of life by quickly recognizing and managing side effects. Individuals differ in terms of the prevalence and risk factors for isotretinoin side effects. Age, gender, dose, duration of treatment, drug interactions, comorbidities, and genetic susceptibility are a few examples of variables that may affect the frequency and intensity of adverse reactions. In severe cases or when side effects become intolerable, dosage modification or treatment interruption may be required. Continued research and vigilance in monitoring are essential to further refine our understanding of isotretinoin’s safety profile and improve patient care while managing acne vulgaris. This comprehensive review has explored the adverse reactions of isotretinoin treatment for acne vulgaris, its incidence, management strategies, and its impact on the patient’s overall quality of life.
